# The effect of high-dose intramuscular epinephrine on the recovery of spontaneous circulation in an asphyxia‐induced cardiac arrest rat model

**DOI:** 10.1186/s12872-021-01917-7

**Published:** 2021-02-25

**Authors:** Daesung Lim, Soo Hoon Lee, Dong Hoon Kim, Changwoo Kang, Jin Hee Jeong, Sang Bong Lee

**Affiliations:** 1 Department of Emergency Medicine, Gyeongsang National University School of Medicine and Gyeongsang National University Hospital, Gangnam-ro 79, Jinju, Gyeongsangnam-Do 52727 Republic of Korea; 2 Department of Emergency Medicine, Gyeongsang National University School of Medicine and Gyeongsang National University Hospital, Samjeongja-ro 11, Seongsan-gu, Changwon, Gyeongsangnam-Do 51472 Republic of Korea

**Keywords:** Asphyxia, Cardiac arrest, Drug administration routes, Epinephrine

## Abstract

**Background:**

Obtaining vascular access can be challenging during resuscitation following cardiac arrest, and it is particularly difficult and time-consuming in paediatric patients. We aimed to compare the efficacy of high-dose intramuscular (IM) versus intravascular (IV) epinephrine administration with regard to the return of spontaneous circulation (ROSC) in an asphyxia-induced cardiac arrest rat model.

**Methods:**

Forty-five male Sprague-Dawley rats were used for these experiments. Cardiac arrest was induced by asphyxia, and defined as a decline in mean arterial pressure (MAP) to 20 mmHg. After asphyxia-induced cardiac arrest, the rats were randomly allocated into one of 3 groups (control saline group, IV epinephrine group, and IM epinephrine group). After 540 s of cardiac arrest, cardiopulmonary resuscitation was performed, and IV saline (0.01 cc/kg), IV (0.01 mg/kg, 1:100,000) epinephrine or IM (0.05 mg/kg, 1:100,000) epinephrine was administered. ROSC was defined as the achievement of an MAP above 40 mmHg for more than 1 minute. Rates of ROSC, haemodynamics, and arterial blood gas analysis were serially observed.

**Results:**

The ROSC rate (61.5%) of the IM epinephrine group was less than that in the IV epinephrine group (100%) but was higher than that of the control saline group (15.4%) (log-rank test). There were no differences in MAP between the two groups, but HR in the IM epinephrine group (beta coefficient = 1.02) decreased to a lesser extent than that in the IV epinephrine group with time.

**Conclusions:**

IM epinephrine induced better ROSC rates compared to the control saline group in asphyxia-induced cardiac arrest, but not compared to IV epinephrine. The IM route of epinephrine administration may be a promising option in an asphyxia-induced cardiac arrest.

## Background

The time from the occurrence of cardiovascular collapse to the return of spontaneous circulation (ROSC) is an important post-arrest prognostic indicator [1–3]. Epinephrine can be helpful in achieving a ROSC by increasing the aortic blood pressure and coronary perfusion pressure [4]. Therefore, epinephrine has been a cornerstone of cardiopulmonary resuscitation (CPR) and advanced cardiac life support since the 1960s.

The most adequate dose, route of administration and time of effective epinephrine administration during cardiac arrest have been investigated. However, vascular access can be challenging during resuscitation following cardiac arrest, and it can be particularly difficult and time-consuming in paediatric patients. Furthermore, vascular access can be distracting when performing high-quality chest compressions, thus adversely affecting haemodynamic status [5, 6]. Intraosseous (IO) or endotracheal epinephrine administration have been introduced as alternative routes [7, 8]. However, IO cannulation also needs special equipment as well as confirmation to exclude the chances of misplacements and the accidental dislodgment of the IO cannula [9–12]. Endotracheal epinephrine administration can be performed only after successful advanced airway management.

 Intramuscular (IM) epinephrine is well established as the initial treatment of choice for systemic anaphylaxis [13]. In the case of anaphylaxis, even if the Blood pressure (BP) is very low at the beginning, IM injection of epinephrine is recommended as the first intervention. The American Heart Association guidelines for CPR and emergency cardiovascular care, and the European Resuscitation Council Guidelines for Resuscitation recommend that cardiac arrest with suspected anaphylaxis should be treated with standard doses of intravenous (IV) or IO epinephrine. However, they support IM epinephrine in cardiac arrest in very limited circumstances. They recommend that IM epinephrine be considered only if cardiac arrest is imminent or has just occurred, unless vascular access by IV or IO is feasible [14, 15]. Except in situations of cardiac arrest following anaphylaxis, few studies have addressed the concerns regarding the impact of IM epinephrine on ROSC during CPR. During standard CPR, peripheral circulation cannot be completely eliminated because the ejection fraction of the left ventricle is maintained above 30% [16–18]. Therefore, there is a possibility that IM injection of epinephrine may be effective during CPR, so this study was planned to assess this possibility. In addition, the control saline group was set up as a comparator arm to determine if ROSC was the effect of the standard CPR itself or of the epinephrine.

Therefore, we aimed to compare the efficacy of IV saline, IV epinephrine, or high-dose IM epinephrine administration with regard to ROSC in an asphyxia-induced cardiac arrest rat model.

## Methods

This study was approved by the Institutional Animal Care and Use Committee (IACUC) of Gyeongsang National University (IACUC reference number: GNU-160,122-R003) and was conducted in accordance with the National Institutes of Health guidelines.

### Animal preparation

Forty-five male Sprague-Dawley rats weighing 300–350 g (Koatech Inc., Peongtaek, Korea) were prepared for this study. The rats were housed in a controlled environment with free access to standard food and water before the experiment. The method of animal preparation has been described previously [19]. Rats were anaesthetized with IM injections of zoletil (30 mg/kg, Virbac, France) and xylazine (15 mg/kg, Bayer, Korea). The rats were intubated using a 14-gauge catheter (BD Insyte TM Autoguard TM, NJ) and connected to a small animal ventilator (tidal volume 0.8 cc/100 g, respiratory rate 55/min, FiO_2_ 0.21; Harvard rodent ventilator model 683, Harvard Apparatus, Holliston, MA). Anaesthesia was maintained by inhalation of 1 % isoflurane through the mechanical ventilator. The minute ventilation was adjusted to ensure a PaCO_2_ between 35 and 40 mm Hg [4.7–5.3 kPa] according to the results of arterial blood gas analysis (ABGA). The rectal temperature was maintained at 36.5–37.5 °C using a heating lamp. IV catheters were inserted into the left femoral artery and vein for blood pressure monitoring, blood sampling for blood gas analysis, and drug administration. BP and heart rate (HR) were recorded and monitored continuously every minute using a Hewlett-Packard Viridia 24 C monitor (Hewlett-Packard, Boeblingen, Germany). A conventional lead II electrocardiogram (ECG) of surface electrodes was also continuously monitored.

### Cardiac arrest and resuscitation

**Fig. 1:**
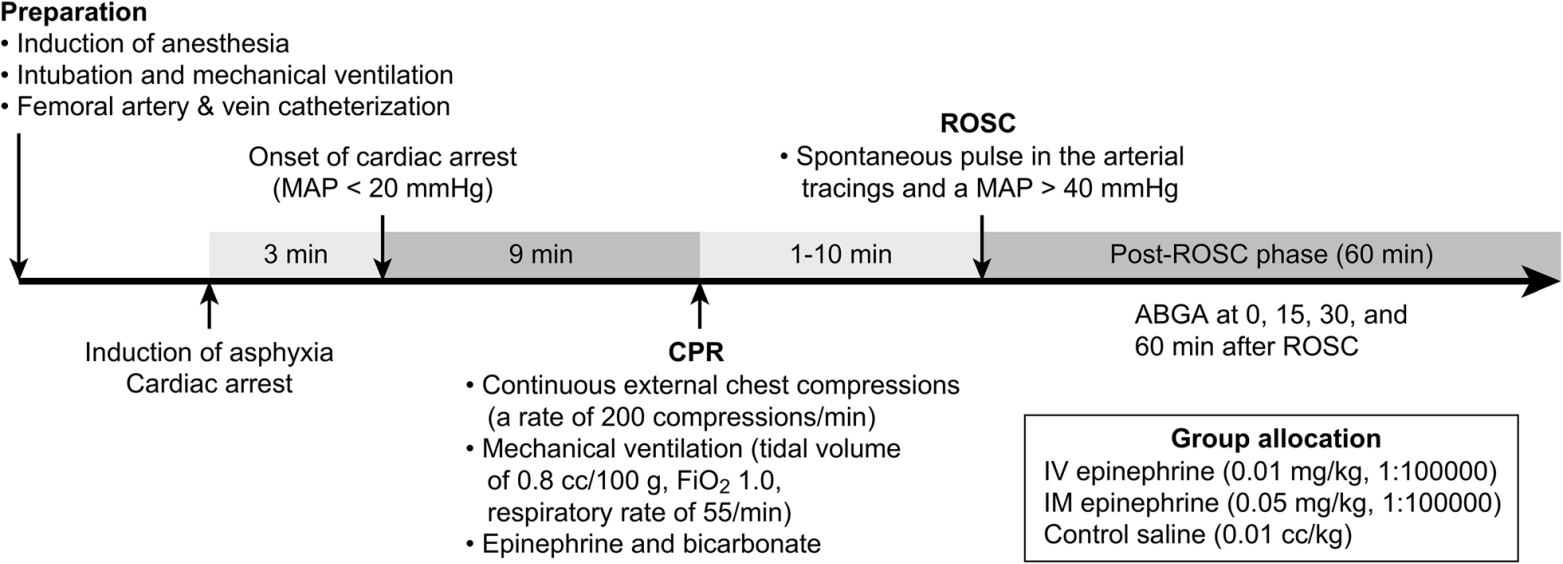
A graphic of the experimental timeline with the experimental protocols

The procedures for the induction of asphyxia-induced cardiac arrest and CPR have been described previously [19]. To induce asphyxia-induced cardiac arrest, vecuronium (0.1 mg/kg, IV) was administered to induce respiratory paralysis and then the mechanical ventilator was disconnected to produce a complete circulatory arrest. A circulatory arrest was defined as a decline of mean arterial pressure (MAP) to 20 mm Hg [2.7 kPa] [20]. In our experiments, 540 s was selected for the no-flow time after circulatory arrest because according to our pilot study, the rate of ROSC without epinephrine administration drops sharply after 8 min and 30 s. In our pilot study, induction of cardiac arrest was achieved between 2 min and 3 min 30 s of asphyxia. To control the duration of the hypoxia, animals were excluded if the induction time of the cardiac arrest was shorter than 2 min or longer than 3 min 30 s. However, the duration of resuscitation was maintained for 15 min taking into account the degree of absorption of the IM agent administered. If ROSC was not achieved even after 15 min of resuscitation, it was defined as a failure of ROSC. After 540 s of circulatory arrest, CPR was performed, which consisted of resuming mechanical ventilation (tidal volume 0.8 cc/100 g, FiO_2_ 1.0, respiratory rate 55/min), administering IV saline (0.01 cc/kg), IV (0.01 mg/kg, 1:100,000) epinephrine, or IM (0.05 mg/kg, 1:100,000) epinephrine and bicarbonate (1.0 mEq/kg), and continuous external chest compressions at a rate of 200 compressions/min using a mechanical thumper (custom made device, compressed air-driven, the rate was set at 200 cycles/min). IV saline, IV epinephrine, or IM epinephrine was given only once during the resuscitation. Sodium bicarbonate was administered after administering IV saline, IV epinephrine, or IM epinephrine after the start of resuscitation. After administration of the IV epinephrine, the catheter was flushed with several drops of normal saline. ROSC was defined as the simultaneous achievement of a spontaneous pulse in the arterial tracings and an MAP above 40 mm Hg [5.3 kPa] for more than 1 min. CPR was continued until ROSC was achieved. We restarted isoflurane 1% through the ventilator after ROSC. After ROSC, the vital signs of the rats were monitored and maintained by mechanical ventilation for 60 min. The surviving rats were euthanized in a carbon dioxide chamber (20L) supplied by compresses gas cylinders after being again anaesthetized with IM injections of zoletil (30 mg/kg) and xylazine (15 mg/kg,). The carbon dioxide flow rate was set at 6 liters/minute to displace 30% of the chamber volume per minute. The surviving rats were left in the chamber for at least 5 min so that complete asphyxia has been attained. The carbon dioxide flow was maintained for 1 minute after apparent clinical death. And then secondary method by decapitation was performed to confirm death prior to disposal of the rat carcass. Figure 1 shows a graphic of the experimental timeline with the experimental protocols.

### Experimental design

The primary outcome for which we were trying to detect an effect was the rates of ROSC. The sample size was calculated using software G-power with an alpha error = 0.05, power(1-beta) = 0.95, effect size = 0.8, degrees of freedom = 2. Therefore, 13 rats were allocated to each group considering an expected loss rate. After asphyxia-induced cardiac arrest, the rats were randomly allocated into one of 3 groups (control saline group (n = 13), IV epinephrine group (n = 13) and IM epinephrine group (n = 13)). The randomization was performed using a random number table by one researcher who prepared the experimental groups, while another researcher performed the animal experiments using the prepared drugs. The quadriceps femoris muscles was selected as the site of IM epinephrine administration, since it is the largest and heaviest muscle in the rat [21]. The dose of IM epinephrine (5 times) was selected based on the maximum dose of IM injection to IV administration used in anaphylaxis.[13] Half of the dose of epinephrine (0.025 mg/kg, 1:100,000) was injected intramuscularly into each hind leg. Sham experiments (2 rats for the control saline group, 2 rats for the IV epinephrine group, and 2 rats for the IM epinephrine group) were conducted following the same experimental procedures, but without inducing a cardiac arrest. The reason we set up the sham group separately from the saline control group in this experiment was to determine whether the decrease in MAP and HR during the post-ROSC observation period were due to the effect of external factors such as an anesthetic or ventilator. Therefore, sham experiments were conducted by setting an additional sham group of 2 rats for each group.

### Measurements and outcomes

Vital signs, including body temperature, blood pressure (systolic, diastolic, mean arterial), HR were measured every minute for 60 min after ROSC in rats subjected to 540 s of cardiac arrest. ABGA was performed serially at 0, 15, 30, 60 min after ROSC.

### Statistical analysis

Parametric variables are reported as the mean ± standard error of the mean (SEM) and nonparametric variables are reported as median (interquartile range). The Kolmogorov–Smirnov test was used to establish normality. Generalized estimation equation (GEE) and repeated-measures analysis of variance (ANOVA) (i.e., blood pressure, HR, body temperature) were performed to compare haemodynamic changes among the groups. Group comparisons (i.e., body weight, ABGA results, induction time of cardiac arrest, resuscitation time, and lactate levels) were performed using one-way ANOVA with the Tukey or Dunnet T3 post hoc analysis, or the Kruskal-Wallis test and Mann-Whitney test with Bonferroni post hoc analysis. P-values less than 0.05 were considered as significantly different. Statistical analyses were performed using SPSS statistical software (version 21.0, IBM, Chicago, IL), and graphs were created with GraphPad Prism 5.0 (GraphPad Software. Inc, La Jolla, CA).

## Results

Table 1 shows the baseline characteristics, including the haemodynamic and arterial blood gas values of the 45 rats investigated. There was no significant differences among the groups in their baseline values. Of the 39 animals in this study, 23 rats had ROSC and 20 of them survived until the 1-h endpoint. The IV epinephrine group (n = 13) had a ROSC rate of 100% (13/13), but only 11 (84.6%) survived until the 1-h endpoint. The IM epinephrine group (n = 13) had a ROSC rate of 61.5% (8/13), and all rats with ROSC survived until the 1-h endpoint. In the control saline group (n = 13), 2 rats of 13 (15.4%) had ROSC, and only 1 of them survived until the 1-h endpoint. All rats (n = 6) in the sham group survived until the 1-h endpoint (Table 2).

**Table 1:** Baseline characteristics including hemodynamic and arterial blood gas values

Variables	IV epinephrine group (n = 13)	IM epinephrine group (n = 13)	Control saline group (n = 13)	Sham group (n = 6)
Body weight	315.4 ± 2.5	318.0 ± 3.1	314.0 ± 3.8	312.5 ± 6.9
*Baseline hemodynamic and arterial blood gas values*				
pH	7.34 ± 0.01	7.34 ± 0.01	7.33 ± 0.01	7.33 ± 0.01
PaCO_2_ (mm Hg)	36.0 ± 1.1	35.9 ± 0.9	35.6 ± 1.3	35.8 ± 1.4
HCO_3_^−^ (mEq L^− 1^)	19.8 ± 0.6	19.4 ± 0.4	19.3 ± 0.6	19.6 ± 0.6
Lactate (mmol L^− 1^)	0.7 ± 0.1	0.9 ± 0.0	0.7 ± 0.1	0.9 ± 0.0
Mean arterial pressure (mm Hg)	96.1 ± 2.3	94.5 ± 1.6	94.2 ± 1.7	92.3 ± 2.2
Heart rate (bpm)	303.8 ± 2.2	304.0 ± 2.4	304.7 ± 2.3	303.7 ± 2.6
Body temperature (℃)	36.8 ± 0.1	37.0 ± 0.1	36.9 ± 0.1	36.9 ± 0.1

*IV* intravenous, *IM* intramuscular, Mean ± Standard Error

**Table 2:** Overview about the animals analyzed in our study

Values	IV epinephrine group (n = 13)	IM epinephrine group (n = 13)	Control saline group (n = 13)	Sham group (n = 6)
ROSC	13/13 (100%)	8/13 (61.5%)	2/13 (15.4%)	
No ROSC	0/13 (0%)	5/13 (38.5%)	11/13 (84.6%)	
Died/no sustained ROSC for 60 min	2/13 (15.4%)	0/13 (0%)	1/13 (7.7%)	0/6 (0%)
Survival rates for 60 min after ROSC	11/13 (84.6%)	8/13 (61.5%)	1/13 (7.7%)	6/6 (100%)

*IV* intravenous, *IM* intramuscular

Table 3 shows the resuscitation and ROSC data. The time to ROSC was longer in the IM epinephrine group than in the IV epinephrine group. This difference may be due to the degree of absorption of the intramuscularly applied epinephrine. Induction time of cardiac arrest, lactate levels and MAP at 60 min after ROSC were similar among the groups. However, lactate levels after ROSC in the IM epinephrine group were significantly lower than those in the IV epinephrine group. The ROSC rates were significantly different among the 3 groups. The ROSC rate (61.5%) of the IM epinephrine group was lower than that in the IV epinephrine group (100%) but much higher than the control saline group (15.4%) (Log-rank test). Two rats from the IV epinephrine group and 1 rat from the control saline group experienced cardiac arrest via ventricular fibrillation at 18 min, 55 min, and right after ROSC (the simultaneous achievement of a spontaneous pulse in the arterial tracings and an MAP above 40 mm Hg for more than 1 minute), respectively. A total of 11 rats in the IV epinephrine group survived to 60 min after ROSC (Tables 2 and 3).

**Table 3:** Values of resuscitation, return of spontaneous circulation and survival

Values	IV epinephrine group	IM epinephrine group	Control saline group	Sham group	*P*-value
Induction time of cardiac arrest (time to MAP < 20 mm Hg) (s)	172.0 ± 6.6	168.15 ± 3.7	168.9 ± 4.7		0.866
Resuscitation time (resuscitation to ROSC) (s)	61.7 ± 2.3	582.8 ± 52.5	441.0 ± 58.0		< 0.001*
Lactate after ROSC (mmol L^− 1^)	8.0 ± 0.4	6.3 ± 0.4	7.0 ± 0.0		0.102
Latate at 60 min after ROSC (mmol L^− 1^)	1.0 ± 0.1	0.9 ± 0.1	0.8 ± 0.0	0.9 ± 0.0	0.475
MAP after ROSC (mm Hg)	61.0 ± 1.8	48.1 ± 1.7	49.0 ± 0.0		0.001†
HR after ROSC	341.2 ± 2.8	280.3 ± 8.0	289.0 ± 0.0		< 0.001†
MAP at 60 min after ROSC (mm Hg)	63.6 ± 1.9	63.6 ± 1.9	53.0 ± 0.0	86.8 ± 0.6	0.255
HR at 60 min after ROSC	206.5 ± 4.8	214.6 ± 9.1	206 ± 0.0	294.7 ± 1.4	0.853
Rates of ROSC	13/13 (100%)	8/13 (61.5%)	2/13 (15.4%)		< 0.001
Survival rates for 60 min after ROSC	11/13 (84.6%)	8/13 (61.5%)	1/13 (7.7%)	6/6 (100%)	< 0.001

*IV* intravenous, *IM* intramuscular, *MAP* mean arterial pressure, *HR* heart rate, *95% CI* 95% confidence interval, *ROSC* return of spontaneous circulation; Mean ± Standard Error; ^∗^*P* < 0.001 compared IV to IM epinephrine, *P* < 0.019 compared IV epinephrine to Control group, *†P* < 0.001 compared IV to IM epinephrine group (post hoc analysis)

**Fig. 2:**
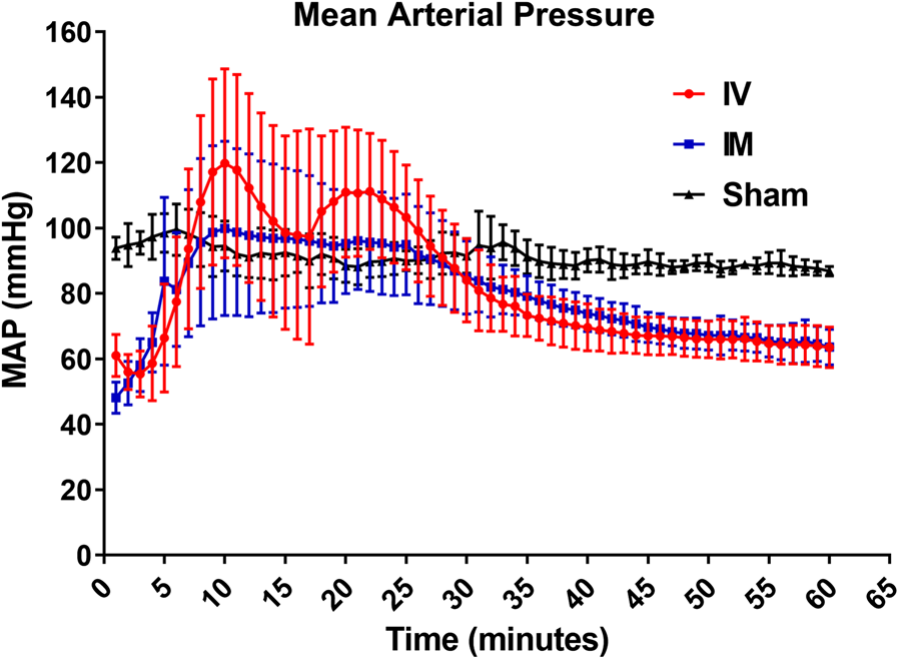
Mean arterial pressure in IM, IV, and sham group for 60 min. *IV* intravenous, *IM* intramuscular

**Fig. 3:**
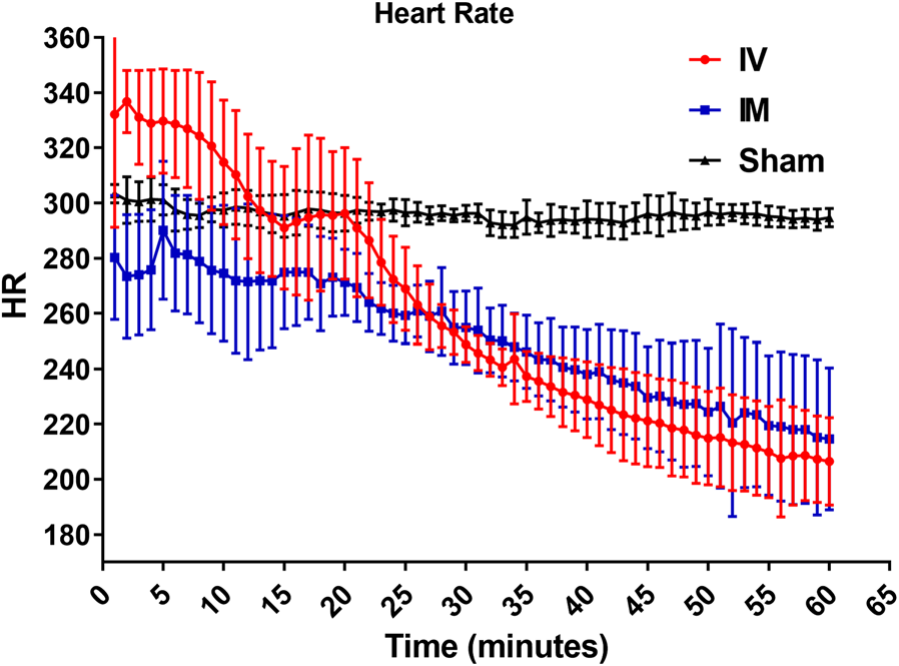
Heart rate in IM, IV, and sham group for 60 min. *IV* intravenous, *IM* intramuscular

The changes in HR and MAP over time in IV, IM epinephrine, and sham groups are shown in Figs. 2 and 3. Table 4 shows the statistical analysis of MAP and HR between IV, IM epinephrine, and sham group. GEE analysis showed that there were no differences (*P* = 0.357, 0.682) in MAP of the IV and IM epinephrine group compared to the sham group, but a difference in HR was noted (*P* = < 0.001, 0.040). To evaluate the change over time between the 3 groups, the interaction between the groups and time was determined. There was a significant difference (*P* = < 0.001, beta coefficient = − 0.47) in the change of MAP over time in the IV epinephrine group compared to the sham group, but no differences (*P* = 0.090, beta coefficient = − 0.22) in the IM epinephrine group was noted. There was a significant difference (both *P* values < 0.001) in the change of HR over time in both IV and IM epinephrine group. HR in the IM epinephrine group (beta coefficient = − 1.11) decreased to a lesser extent than in the IV epinephrine group (beta coefficient = − 2.20) with time.

**Table 4:** Statistical analysis of mean arterial pressure and heart rate among intravenous, intramuscular epinephrine, and Sham Group

Variables		MAP				HR			
		Estimate	95% CI		*P*-value	Estimate	95% CI		*P*-value
Group	Sham	Ref.				Ref.			
	IV	2.43	− 9.20	14.06	0.682	27.01	16.18	37.85	< 0.001
	IM	− 5.65	− 17.65	6.36	0.357	− 11.70	− 22.87	− 0.53	0.040
Time		− 0.13	− 0.21	− 0.06	< 0.001	− 0.08	− 0.16	0.00	0.051
Time* Group	Sham	Ref.				Ref.			
	IV	− 0.47	− 0.64	− 0.29	< 0.001	− 2.20	− 2.45	− 1.96	< 0.001
	IM	− 0.22	− 0.48	0.04	0.090	− 1.11	− 1.46	− 0.76	< 0.001

*IV* intravenous, *IM* intramuscular, *MAP* mean arterial pressure, *HR* heart rate, *95% CI* 95% confidence interval

## Discussion

The main finding of this study was that the ROSC rate in the IM epinephrine group was lower than in the IV epinephrine group but much higher than that in the control saline group. These experimental results confirm the efficacy of an IM injection of epinephrine as an alternative route of administration in asphyxia-induced cardiac arrest.

Non-shockable rhythms predominate in both paediatric and adult out-of-hospital cardiac arrest (OHCA). Early and timely administration of epinephrine after the onset of cardiac arrest with a non-shockable rhythm has been recommended in the most recent CPR guidelines. The time to the first epinephrine injection was a predictive factor of ROSC in several studies involving cardiac arrest including animal trials [22, 23]. Early administration of epinephrine improves outcomes via two mechanisms: (1) shortening the duration of a cardiac arrest, and (2) minimizing post-ROSC complications associated with delayed administration. However, vascular access can be challenging during cardiac arrest since it can be problematic and time-consuming to perform an IV cannulation during CPR. Zuercher et al. [24] showed that early IO epinephrine shortened the time to ROSC and consequently resulted in a better 24-h survival rate than delayed IV epinephrine in pigs. However, IO cannulation also needs special equipment, and accidental misplacements or dislodgment of the IO cannula are major concerns [9–12]. In cases such as cardiac arrest, the determination of an easily accessible and reliable alternative route of administration is required.

Although multiple studies revealed that epinephrine increases ROSC in OHCA, the influence on long-term survival (survival to discharge with good neurologic outcomes) is questionable [25, 26]. However, the timing of epinephrine administration after the onset of cardiac arrest, rather than the negative effects associated with epinephrine, can be more influential for long-term survival and the neurologic outcomes of cardiac arrest victims. According to a literature review of studies involving out-of-cardiac arrest patients, the mean time to the first IV drug administration was 19.4 min, whereas in animal studies it was 9.5 min [23, 27]. Therefore, the importance of vascular access has been emphasized to shorten the time to administration of a drug. IM epinephrine administration is well established and widely used in treating patients with severe anaphylaxis [13]. Auto-injectors have been well established and safely used in the field of severe anaphylaxis. Specially manufactured epinephrine auto-injectors (EpiPen) can be applied easily, even by nonprofessionals if required. The IM route will have several advantages during resuscitation. First, the IM route does not require special equipment, it is relatively easy to perform in a short period of time, and it has a greater margin of safety than the IV route. However, the effects of IM epinephrine administration need further investigations in cases of cardiac arrest. If the effect of IM epinephrine is also reproducible in cardiac arrest, it would be reasonable to provide prefilled epinephrine syringes, as ‘High-Dose-Epi’-auto-injectors. IM administration of epinephrine would then be available similar to an automated external defibrillator for laypersons where training on IV/IO and equipment availability is not reasonable.

In anaphylaxis, the peak serum epinephrine concentration depends on the injection site. IM injections given in the vastus lateralis muscle had a five-fold higher peak serum epinephrine concentration than those following injection in the deltoid muscle [28]. Unlike in anaphylaxis, wherein adequate blood flow continues to the muscles, in asystole, blood flow to the muscles is compromised, and any deposition of drug in the muscle may not achieve circulation. Nevertheless, a previous study using a 10-fold higher dose for IM than IV administration indicated predictable systemic absorption of IM epinephrine during CPR in piglets. In these studies, the tongue was chosen as the IM injection site [29, 30]. However, these studies did not confirm the impact of IM epinephrine administration on ROSC and survival during CPR in cases of acute asphyxia-induced circulatory arrest, primarily because of the many drop-outs (i.e., ROSC without epinephrine administration after cardiac arrest). The unsatisfactory results seen in these studies were assumed to be caused by a relatively short no-flow time (8 min), as well as the IM injection site (tongue). Therefore, in our experiments, the quadriceps femoris muscle was selected as the IM injection site, as it is the largest and heaviest muscle in the rat [21]. In addition, a 5-fold higher dose for IM compared to the IV administration was used according to previous recommendations for the treatment of anaphylaxis in humans. In this study, the reason for a significantly higher rate of ROSC in the IM epinephrine group than the control saline group may be due to epinephrine being systemically absorbed in predictable amounts during CPR.

This study has several limitations. This study involved a small sample size of a rodent model of asphyxia-induced cardiac arrest. Therefore, it cannot be generalized to larger animals or humans and to cardiac arrests due to other aetiologies, although asphyxia-induced circulatory arrest is a main indication for resuscitation, and rats are an established model in resuscitation research. The survival rate after cardiac arrest from asphyxia is very low and it is well-known to cause severe hypoxic brain damage in survivors. Therefore, animal studies on the effect of IM epinephrine based on brain histopathologic findings should be performed in the future. In addition, whether IM epinephrine can be applied in other forms of cardiac arrest has to be investigated in further studies. Furthermore, the variability of resorption of the drug from the muscle is likely related to several conditions (hypovolemic shock, asphyxia, sepsis) that lead to cardiac arrest. A larger study population with measurement of serum epinephrine levels is needed to confirm these preliminary results and to define the adequate dose of IM epinephrine.

## Conclusions

IM epinephrine induced better ROSC rates compared to the control saline group in asphyxia induced cardiac arrest, but not compared to IV epinephrine. The IM route of epinephrine administration may be a promising option in asphyxia induced cardiac arrest without IV or IO access.

## Data Availability

The datasets used and/or analysed during the current study are available from the corresponding author on reasonable request.

## Abbreviations

ROSC: Return of spontaneous circulation
CPR: Cardiopulmonary resuscitation
IO: Intraosseous
IM: Intramuscular
IV: Intravenous
ABGA: Arterial blood gas analysis
BP: Blood pressure
HR: Heart rate
ECG: Electrocardiogram
MAP: Mean arterial pressure
SEM: Standard error of the mean
GEE: Generalized estimation equation
ANOVA: Analysis of variance
OHCA: Out-of-hospital cardiac arrest
